# Clinical predictors of causative radiographic findings in adults with acute onset diplopia

**DOI:** 10.3389/fneur.2024.1470805

**Published:** 2024-11-27

**Authors:** Yubin Son, Hie Bum Suh, Hee-young Choi, Michelle T. Cabrera, Hyeshin Jeon

**Affiliations:** ^1^Department of Ophthalmology, School of Medicine, Pusan National University, Yangsan, Republic of Korea; ^2^Biomedical Research Institute, Pusan National University Hospital, Busan, Republic of Korea; ^3^Department of Radiology, Pusan National University Hospital, Busan, Republic of Korea; ^4^Department of Ophthalmology, University of Washington, Seattle, WA, United States; ^5^Division of Ophthalmology, Seattle Children’s Hospital, Seattle, WA, United States

**Keywords:** strabismus, binocular, diplopia, cranial nerve palsy, diagnosis, MRI

## Abstract

**Objective:**

This study aimed to investigate the clinical factors that predict abnormal radiographic findings in adults with acute onset binocular diplopia.

**Methods:**

Medical records of consecutive patients aged >20 years who presented with acute binocular diplopia were retrospectively reviewed. Patients were divided into positive and negative groups according to radiographic findings. Demographic and clinical characteristics were compared. The risk factors for positive radiographic findings were investigated, and the area under the receiver operating characteristic curve (AUC) was calculated.

**Results:**

Among 242 patients (145 males and 97 females), 44 (18.2%) were in the positive group and 198 (81.8%) in the negative group. Patients in the positive group were older (*p* = 0.005) and had more vasculopathic risk factors (*p* = 0.038). Severe duction limitation (>50% reduction in motility) was present in 90.9% of patients in the positive group and 56.1% in the negative group (*p* < 0.001). Abnormal slit lamp findings and pupillary exam abnormalities were also more frequent in the positive group than in the negative group (*p* = 0.027 and *p* = 0.036, respectively). Older age, higher intraocular pressure, abnormal slit-lamp findings, exophthalmos, and duction limitation were identified as risk factors for positive radiographic findings. A predictive model generated an AUC of 0.772.

**Conclusion:**

Older age and vasculopathic risk factors were associated with underlying radiographic pathologies, supporting the recommendation that neuroimaging should not be delayed in those patients. Careful ophthalmic evaluations may guide diagnosis and decision-making for immediate neuroimaging in cases of diplopia.

## 1 Introduction

Acute-onset strabismus with associated binocular diplopia in adults is not uncommon and has diverse etiologies. Although cranial nerve palsy is the most common cause, other conditions such as orbital disease, neuromuscular junction disorder, and supranuclear disease can also lead to acute binocular diplopia (1, 2). Moreover, serious underlying conditions such as intracerebral aneurysm, brain tumor, pituitary apoplexy, and stroke can pose significant threats to both life and vision (3–6). Therefore, timely and appropriate brain imaging is crucial to guide effective management.

Contrast-enhanced magnetic resonance imaging (MRI) is considered the gold standard for diagnosing the cause of the diplopia (7, 8); however, limitations such as time and cost constraints exist. Given the wide range of potential causes of diplopia, physicians need more nuanced guidance on when to perform timely imaging. Here, we aimed to investigate the risk factors for abnormal radiographic findings in adult patients with acute-onset diplopia.

## 2 Materials and methods

This retrospective study was approved by the Institutional Review Board of Pusan National University Hospital (IRB number 2202-022-111). The requirement for informed consent was waived because of the study’s retrospective nature.

### 2.1 Patient inclusion

The medical records of consecutive patients aged >20 years who presented with acute binocular diplopia between 2010 and 2020 at the ophthalmology or neurology clinic of a single tertiary medical center were retrospectively reviewed. Patients were included if they had undergone a comprehensive ophthalmic assessment and systemic workup, including MRI and laboratory testing. Patients with a history of orbital surgery, strabismus surgery, or known systemic diseases that could influence ocular alignment, such as thyroid eye disease or myasthenia gravis, were excluded. Patients presenting with acute diplopia accompanied by new-onset neurological symptoms were not excluded.

### 2.2 Ophthalmic assessment

As part of standard care, all patients underwent a complete ophthalmic examination. This examination was conducted by a single, fellowship-trained neuro-ophthalmologist and strabismus surgeon. The ocular deviation angle was measured by prism and alternate cover testing in the primary and secondary gaze positions, and in the up, down, right, left, and tilted gaze positions. Alignment measurements were performed at both 6 and 1/3 m. Additionally, ductions and versions were assessed to evaluate eye movement limitations. Eye movement limitations were classified into three categories based on the subjective assessment of the ophthalmologist: mild, with a limitation of <50% of the normal range; severe, with a limitation of >50% of the normal range; and absent, without ocular motility limitation. Intraocular pressure was measured. Slit lamp and fundus examinations were performed to examine the structural ophthalmic findings. The presence of ptosis or exophthalmos was also assessed. Exophthalmos was examined using Hertel exophthalmometry, and significant exophthalmos was defined as a difference of at least 2 mm between the two eyes.

### 2.3 MRI assessment

A 1.5-T MRI scanner (Siemens, Erlangen, Germany) was used. Each patient underwent MRI imaging that consisted of T2-weighted sequences, and sagittal and coronal T1-weighted sequences, performed before and after gadolinium contrast. The images were evaluated for findings relevant to the pathophysiology of diplopia.

### 2.4 Statistical analysis

Patients were divided into positive and negative groups according to radiographic findings. The clinical characteristics of the two groups were compared using the Mann–Whitney U test or paired *t*-test for continuous variables and the Chi-square test or Fisher’s exact test for frequency variables. The risk factors for positive radiographic findings were investigated using binary logistic regression analysis, and the area under the receiver operating characteristic curve (AUC) was calculated. Data were analyzed using SPSS (version 19.0, Chicago, IL, United States) and R software (R Foundation for Statistical Computing, Vienna, Austria). Statistical significance was set at *p* < 0.05.

## 3 Results

A total of 242 patients (145 males and 97 females) were included in this study. Forty-four (18.2%) and 198 (81.8%) patients were included in the positive and negative groups, respectively. Positive radiographic findings included intracranial aneurysm in 6 (13.6%), brain infarction in 1 (2.3%), brain tumor in 9 (20.5%), encephalitis in 1 (2.3%), meningitis in 2 (4.5%), cranial neuritis in 1 (2.3%), carotid-cavernous fistula in 1 (2.3%), orbital disease including orbital mass in 2 (4.5%), idiopathic orbital inflammation (IOI) in 10 (22.7%), and thyroid eye disease (TED) in 11 (25.0%) patients (Table 1).

**Table 1:** Abnormal radiographic findings in patients presenting with acute diplopia.

Diagnosis	Number of patients (%)
Total patients	44 (100)
Intracranial aneurysm	6 (13.6)
Brain infarction	1 (2.3)
Brain tumor	9 (20.5)
Encephalitis	1 (2.3)
Meningitis	2 (4.5)
Cranial neuritis	1 (2.3)
Carotid-cavernous fistula	1 (2.3)
Orbital mass	2 (4.5)
Idiopathic orbital inflammation	10 (22.7)
Thyroid eye disease	11 (25.0)

Table 2 presents the diagnosis of the patients with diplopia. Cranial nerve palsy (124/242 patients, 51.2%) was the most common cause of diplopia. All were single isolated cranial nerve palsies. There were 23 (18.5%) patients with oculomotor palsy, 57 (46.0%) with trochlear palsy, and 44 (35.5%) with abducens palsy. Among these cases, positive radiographic findings were found in 18 (14.5%) patients, with oculomotor palsy having the highest rate (6/23, 26%), followed by abducens palsy (10/44, 22.7%) and trochlear palsy (2/57, 3.5%). Other etiologies include comitant strabismus (32.6%), strabismus caused by orbital disease (12.0%), ocular myasthenia gravis (2.1%), and supranuclear palsy (0.4%).

**Table 2:** Clinical diagnoses of patients with and without radiographic findings.

	Total	Negative group^*^	Positive group^†^
Cranial nerve palsy	124 (51.2)	106 (53.5)	18 (40.9)
Oculomotor nerve palsy	23 (18.5)	17 (16.0)	6 (33.3)
Trochlear nerve palsy	57 (46.0)	55 (51.9)	2 (11.1)
Abducens nerve palsy	44 (35.5)	34 (32.1)	10 (55.6)
Comitant strabismus	79 (32.6)	76 (38.4)	3 (6.8)
Strabismus caused by orbital disease	29 (12.0)	8 (4.0)	21 (47.7)
Ocular myasthenia gravis	5 (2.1)	5 (2.5)	0 (0)
Supranuclear palsy	1 (0.4)	0 (0)	1 (2.3)
Others^‡^	4 (1.7)	3 (1.5)	1 (2.3)

All data presented as number of patients (%).

* Patients without abnormal radiographic findings that could contribute to diplopia.

† Patients with abnormal radiographic findings that could contribute to diplopia.

‡ Others includes elevation palsy, heavy eye syndrome, and Duane retraction syndrome.

Patients were older (63.8 ± 13.5 vs. 56.8 ± 17.7 years, *p* = 0.005) and had more frequent vasculopathic risk factors including diabetes mellitus, hypertension or hypercholesterolemia (25/44 [56.8%] vs. 76/198 [38.4%], *p* = 0.038) in the positive group than in the negative group. Patients in the positive group were more likely to take medications for underlying systemic comorbidities, including diabetes mellitus, hypertension, hypercholesterolemia, and others, than those in the negative group (*p* = 0.038). Other ophthalmic outcomes were found to be different between the groups. Specifically, duction limitations were present in 90.9 and 56.1% of patients in the positive and negative groups, respectively (*p* < 0.001). Abnormal slit lamp findings that may be affected by the underlying disease were also more frequent in the positive group than in the negative group (p < 0.001), including conjunctival injection in 21 patients (8.7%), chemosis in 7 (2.9%), and episcleral injection in 2 (0.8%). Abnormalities in the pupillary exam were identified in two patients (0.8%) in the positive group and none (0%) in the negative group (*p* = 0.036); specifically, these patients presented with mid-dilated and fixed pupils. Assessing results of the automated visual field examinations, there were no significant differences between the two groups (Table 3).

**Table 3:** Comparison of clinical characteristics of patients with acute-onset diplopia with and without radiographic findings.

	Negative group^*^	Positive group^†^	*p* value
Demographics			
Age (years, mean ± SD)	56.8 ± 17.7	63.8 ± 13.5	0.005
Sex (M: F)	119:79	26:18	1
Vasculopathic risk factors [no. of patients, (%)]	76(38.4)	25 (56.8)	0.038
Diabetes mellitus	46 (23.5)	11 (25.0)	0.984
Hypertension	48 (24.2)	17 (38.6)	0.078
Hypercholesterolemia	15 (7.6)	5 (11.4)	0.601
Systemic medication	98 (49.5)	30 (68.2)	0.038
Ophthalmic features			
Degree of ocular movement limitation			
Absent ^‡^	87 (43.9)	4 (9.1)	<0.001
Mild ^§^	84 (42.4)	26 (59.1)	0.137
Severe^Π^	27 (13.7)	14 (31.8)	
Abnormal slit lamp finding	15 (8.2)	12 (27.3)	<0.001
Ptosis	23 (11.6)	8 (18.2)	0.353
Exophthalmos^#^	13 (6.6)	9 (20.5)	0.009
Abnormal light reflex	0 (0.0)	2 (4.5)	0.036
Visual field defect^**^	22 (51.2)	12 (57.1)	0.854

All data presented as number of patients (%) unless otherwise specified. SD, Standard deviation. M:F, male: female.

* Patients without abnormal radiographic findings that could contribute to diplopia.

† Patients with abnormal radiographic findings that could contribute to diplopia.

‡ Ocular movement defect was not observed.

§ Limitation of less than 50% of the normal range.

Π Limitation of greater than 50% of the normal range.

# Defined as at least a 2 mm difference between the two eyes.

** Among patients who underwent automated visual field tests.

Among the patients with cranial nerve palsy, 18 of 124 (14.5%) patients presented with positive radiographic findings. They were older (71.4 ± 7.6 vs. 63.7 ± 13.7 years, *p* = 0.001) and presented with more frequent pupil involvement (2/18 [11.1%] vs. 0/106 [0%], *p* = 0.014) compared to patients without positive radiographic findings. Both patients with pupil involvement had oculomotor palsy (caused by herpetic neuritis and intracranial aneurysm, respectively) (Table 4).

**Table 4:** Comparison of clinical characteristics of patients with cranial nerve palsy with and without radiographic findings.

	Negative group^*^	Positive group^†^	*p* value
Demographics			
Age (years, mean ± SD)	63.7 ± 13.7	71.4 ± 7.6	0.001
Sex (M: F)	72:34	10:8	0.450
Vasculopathic risk factors (n. of patients, (%))	57 (53.8)	12 (66.7)	0.446
Diabetes mellitus	37 (34.9)	8 (44.4)	0.608
Hypertension	38 (35.8)	9 (50)	0.378
Hypercholesterolemia	9 (8.5)	1 (5.6)	1.000
Ophthalmic features			
Degree of ocular movement limitation			
Absent ^‡^	24 (22.6)	1 (5.6)	0.079
Mild ^§^	54 (50.9)	11 (61.1)	0.363
Severe^Π^	28 (26.4)	6 (33.3)	
Abnormal slit lamp finding	8 (7.5)	3 (16.7)	0.200
Ptosis	18 (17.0)	3 (16.7)	1.000
Exophthalmos^#^	5 (4.7)	3 (16.7)	0.165
Abnormal light reflex	0 (0)	2 (11.1)	0.014
Visual field defect^**^	12 (57.1)	2 (66.7)	0.426

All data presented as number of patients (%) unless otherwise specified. SD, Standard deviation. M:F, male: female.

* Patients without abnormal radiographic findings that could contribute to diplopia.

† Patients with abnormal radiographic findings that could contribute to diplopia.

‡ Ocular movement defect was not observed.

§ Limitation of less than 50% of the normal range.

Π Limitation of greater than 50% of the normal range.

# Defined as at least a 2 mm difference between the two eyes.

** Among patients who underwent automated visual field tests.

Comitant strabismus (79/242 patients, 32.6%) was the second most common cause of diplopia, with exotropia in 27 (34.2%), esotropia in 49 (62.0%), small vertical strabismus in 1 (1.3%), and esotropia combined with small vertical strabismus in 2 (2.5%) patients. Among those with comitant strabismus, only 3 (3.8%) patients had positive radiographic findings (all had esotropia).

**Figure 1:**
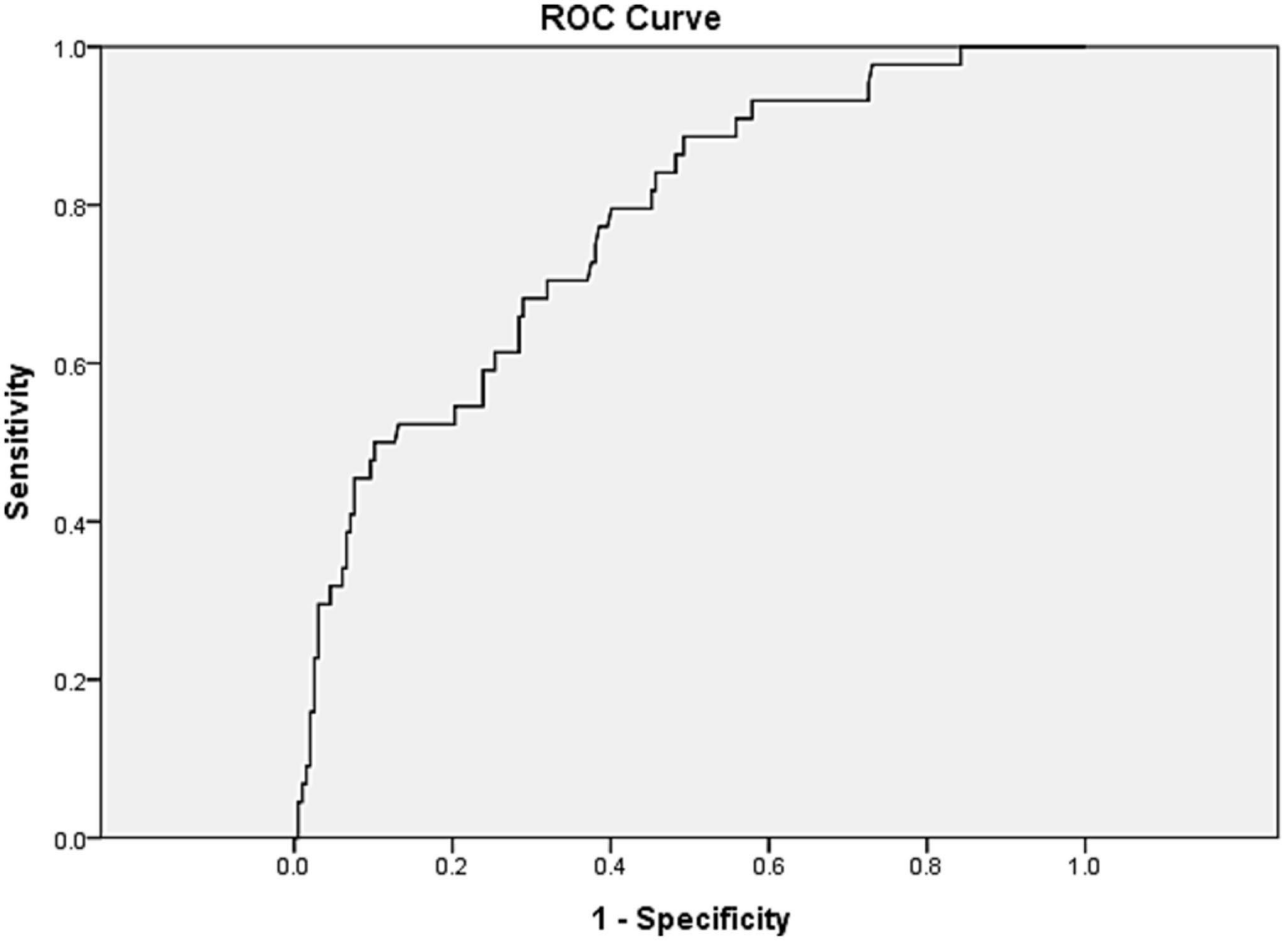
Receiver operating characteristic curve for a model to predict positive radiographic findings in patients with diplopia. The *x*-axis represents 1-specificity while the *y*-axis represents sensitivity. Risk factors in the model included older age, intraocular pressure, abnormal slit lamp findings, exophthalmos, and ocular duction limitations. ROC, Receiver operating characteristic.

Univariate analysis revealed that older age, higher intraocular pressure, abnormal slit-lamp findings, exophthalmos, and severe duction limitations were risk factors for positive radiographic findings (Table 5). A predictive model was created comprising age, intraocular pressure, abnormal slit-lamp findings, exophthalmos, and ocular duction limitations. The AUC was calculated to be 0.772 (Figure 1).

**Table 5:** Logistic regression analysis for clinical factors associated with positive radiographic findings.

	Exp(B)^1^	95% confidence interval		*p* value
		Lower	Upper	
Demographics				
Age	1.023	1.002	1.044	0.035
Sex	1.043	0.536	2.028	0.902
Vasculopathic risk factor^2^ ≥ 1	1.886	0.976	3.643	0.059
Ophthalmologic findings				
Visual acuity	1.695	0.325	8.835	0.531
Intraocular pressure	1.129	1.039	1.228	0.004
Abnormal slit lamp finding	2.595	1.184	5.689	0.017
Presence of ptosis	1.372	0.55	3.42	0.498
Presence of exophthalmos^3^	4.559	1.825	11.387	0.001
Abnormal pupillary light reflex	7.6*10^9^	0		0.999
Severe ocular movement limitation	1.597	1.236	2.019	<0.001

1 Exponentiation of the B coefficient, which is an odds ratio.

2 Include diabetes, hypertension, and hypercholesterolemia.

3 Defined as at least a 2 mm difference between the two eyes.

## 4 Discussion

In this study, 18.2% of patients with acute-onset diplopia who underwent brain MRI had underlying radiographic lesions, many requiring prompt treatment. These included aneurysm (13.6%), IOI (22.7%), brain tumor (20.5%), brain infarction (2.3%), encephalitis (2.3%), meningitis (4.5%), carotid-cavernous fistula (2.3%), and orbital mass (4.5%). With the exception of three meningioma cases, which were managed with periodic follow-up, all conditions required treatment. Ophthalmic findings in these patients differed from those of patients without radiographic abnormalities. Univariate analysis indicated that older age, elevated intraocular pressure, abnormal slit-lamp findings, exophthalmos, and ocular duction limitations were associated with radiographic findings linked to specific disorders.

Cranial nerve palsy is one of the most common causes of acute onset diplopia. Ischemia is the most common etiology, and inflammation, neoplasm, and trauma are also frequent culprits (8–10). Previous studies recommend limited indications for immediate imaging in acute isolated cranial neuropathy cases (11, 12). In the study by Murchison et al. (11), only four of 93 patients (4.3%) over 50 years old with isolated cranial neuropathy (3, 4, 6) had MRI-detected lesions, most of which did not impact treatment or prognosis. Based on their findings, they recommended performing MRI in patients with isolated cranial nerve palsies if they met any of the followings: age < 50 years, history of cancer, presence of additional neurological signs, pupil involvement, partial third cranial nerve palsy, or lack of resolution 3 months after the initial visit. Some have also used vascular risk factors, such as diabetes or hypertension, to support underlying ischemia as the likely cause of acute cranial nerve palsy, thereby suggesting that immediate neuroimaging may be unnecessary in those cases (12).

In contrast, Tamhankar et al. (7) reported that 16.5% of patients aged ≥50 years with isolated cranial nerve palsy demonstrated underlying pathologies other than microvascular ischemia, many of which had important implications for timely intervention. After excluding patients with oculomotor palsies and those with giant cell arteritis, they reported that the incidence of radiographic causative lesions other than presumed microvascular for isolated trochlear and abducens nerve palsies was 4.7% among patients with vasculopathic risk factors. Our findings align with theirs, showing that oculomotor nerve palsy had the highest frequency. They also suggested immediate diagnostic imaging for all cranial nerve palsy patients, since distinguishing ischemic causes from serious intracranial diseases without immediate neuroimaging proved challenging (6, 13).

In this study, half of the patients were diagnosed with a single isolated cranial nerve palsy, and 13.8% had positive radiographic findings, which exceeded the rates reported by Murchison et al. (11) Notably, when considering only patients over 50 years of age, consistent with the criterion established in Murchison’s study (11), the rate of positive findings increased to 17.3% (18 of 105 patients), thereby challenging the use of younger age as a criterion for immediate neuroimaging. Furthermore, positive radiographic findings were *more* frequent in patients with vascular risk factors in our study. Similarly, Tamhankar et al. (7) found that 61% (11/18) of patients with primary causes of cranial nerve palsy on brain imaging had vasculopathic risk factors. Therefore, we argue against using vascular risk factors or older age as reasons to forego neuroimaging. Pupil involvement was more frequent in the patients with positive radiographic findings, which was in line with the result of the other studies.

The most frequent disease among the patients with associated radiographic findings was orbital disease, including TED and IOI in our study. These patients commonly present with a history of thyroid dysfunction and/or typical exophthalmos and eyelid changes; therefore, these conditions are often diagnosed clinically before imaging (14). Nonetheless, the clinical presentations of these disorders may be subtle (15). In this study, clinically significant exophthalmos was a significant risk factor for positive radiographic findings. Therefore, the examination of orbital signs, including exophthalmometry, may be helpful in the diagnosis of diplopia.

Among patients with comitant strabismus, only 3.8% showed positive MRI findings, all of whom presented with esotropia. Esotropia is a common form of acute comitant strabismus in adults (16). A recent report noted that its frequency is increasing (17). Arnold–Chiari syndrome, brain tumors, or increased intracranial pressure should be suspected (18, 19). Previous studies have shown that patients with acute comitant strabismus without a history of strabismus, occlusion therapy, nystagmus, or an inability to restore binocularity are more likely to have underlying intracranial diseases (20).

Our study has several limitations. First, this was a retrospective study that included patients who visited an ophthalmology clinic and underwent MRI due to diplopia. Since participants were excluded if an appropriate ophthalmological evaluation was missing, patients with severe neurological signs or symptoms may have been excluded, leading to selection bias. Second, the diseases represented in this study were broad, and a risk factor analysis for each disease was not performed. Third, the resultant MRIs were evaluated by a single radiologist, without a second radiologist’s confirmation.

In conclusion, 18.2% of the patients with acute-onset diplopia in this study demonstrated various abnormal MRI findings. Vasculopathic risk factors and older age were associated with underlying radiographic pathologies and should not be used as criteria for delayed neuroimaging. A thorough ophthalmologic evaluation, including strabismus examination, examination for anterior segment abnormalities, intraocular pressure, and exophthalmometry, may help ensure a timely diagnosis and facilitate appropriate management for these patients.

## Data Availability

The original contributions presented in the study are included in the article/supplementary material, further inquiries can be directed to the corresponding author.
